# Association between serum levels of interleukin-6 on ICU admission and subsequent outcomes in critically ill patients with acute kidney injury

**DOI:** 10.1186/s12882-019-1265-6

**Published:** 2019-03-01

**Authors:** Takashi Shimazui, Taka-aki Nakada, Yoshihisa Tateishi, Taku Oshima, Tuerxun Aizimu, Shigeto Oda

**Affiliations:** 1 Department of Emergency and Critical Care Medicine, Chiba University Graduate School of Medicine, 1-8-1 Inohana, Chuo-ku, Chiba, 260-8670 Japan; 2 Graduate School of Engineering, Chiba University, 1-33 Yayoi-cho, Inage-ku, Chiba, 263-8522 Japan

**Keywords:** AKI, Anuria, Interleukin-6, Inflammation, Mortality, Renal recovery

## Abstract

**Background:**

Exacerbated inflammatory response is considered one of the key elements of acute kidney injury (AKI). Interleukin-6 (IL-6) is an inflammatory cytokine that plays important roles in the inflammatory response and may be useful for predicting the clinical outcomes in patients with AKI. However, supporting evidence adapted to the current KDIGO criteria is lacking.

**Methods:**

AKI patients admitted to the ICU between Jan 2011 and Dec 2015 were retrospectively screened. Patients were assigned to three groups by admission IL-6 tertiles. Associations between IL-6 on ICU admission and in-hospital 90-day mortality, short-term/long-term renal function were analyzed.

**Results:**

Patients (*n* = 646) were divided into low (1.5–150.2 pg/mL), middle (152.0–1168 pg/mL), and high (1189-2,346,310 pg/mL) IL-6 on ICU admission groups. Patients in the high IL-6 group had higher in-hospital 90-day mortality (low vs. middle vs. high, *P* = 0.0050), lower urine output (low vs. middle vs. high, *P* < 0.0001), and an increased probability of persistent of anuria for ≥12 h (low vs. middle vs. high, *P* < 0.0001) within 72 h after ICU admission. In contrast, the high IL-6 group had a lower incidence of persistent AKI at 90 days after the ICU admission in survivors (low vs. middle vs. high, *P* = 0.013).

**Conclusions:**

Serum levels of IL-6 on ICU admission may predict short-term renal function and mortality in AKI patients and were associated with renal recovery in survivors.

## Background

Acute kidney injury (AKI) is frequently observed in critically ill patients treated in the intensive care unit (ICU) and recognized as a significant risk factor of mortality [1, 2]. AKI is diagnosed using the Kidney Disease: Improving Global Outcomes (KDIGO) criteria based on changes in creatinine and urine output. Renal replacement therapy (RRT) is indicated when advanced AKI is identified [3–5]. Short-term kidney function, such as persistent oliguria or anuria, becomes a key determinant for initiating RRT [6]. However, the optimal timing to initiate RRT remains controversial. A better prediction of AKI can improve the quality of the acute phase management of AKI and may contribute to improving long-term outcomes of kidney function, i.e. recovery from AKI or reducing mortality. Among the various factors that contribute to the development of AKI, an exacerbated inflammatory response is considered one of the key elements and is supposedly associated with clinical outcomes [7, 8].

Cytokines play important roles in the inflammatory response and may induce organ dysfunction when released in excess [9, 10]. Interleukin-6 (IL-6) is an inflammatory cytokine that is elevated in the plasma soon after an insult and peaks while still in the acute phase of the critical illness [11, 12]. Serum level of IL-6 is associated with clinical outcome or organ dysfunction severity in critically ill patients [13–15] and may be useful for predicting the development of AKI [16, 17]. IL-6 is also associated with mortality in acute renal failure (ARF) [18–20] and may be useful for predicting the clinical outcomes in patients with AKI, but supporting evidence adapted to the current KDIGO criteria are lacking.

We hypothesized that serum level of IL-6 on ICU admission is a useful tool for predicting the clinical outcomes in patients with AKI. To test this hypothesis, we sought to investigate the associations between serum levels of IL-6 and the mortality, morbidity, and outcomes in patients with AKI in the ICU.

## Methods

### Patients

This retrospective observational study included a cohort of 8715 patients admitted to the medical/surgical ICU of Chiba University Hospital between January 2011 and December 2015. Adult (≥18 years of age) AKI patients without a previous diagnosis of end-stage renal disease (ESRD) requiring dialyzes who stayed in the ICU for ≥48 h were enrolled. Patients were excluded if baseline data (serum creatinine, urine output, body weight) were missing, an AKI diagnosis within 24 h after ICU admission was lacking, or data on serum IL-6 levels on ICU admission were lacking.

### Data collection and definition

Baseline characteristics consisting of age, sex, body weight, comorbidities (chronic kidney disease [CKD], hypertension [HT], diabetes mellitus [DM]), severity scores (Acute Physiology and Chronic Health Evaluation [APACHE] II score, Sequential Organ Failure Assessment [SOFA] score), and etiologies of AKI (sepsis, cardiovascular disease, hypovolemia, severe acute pancreatitis, major surgery, hepatic failure, urinary tract obstruction, drug-induced, and other) were retrieved. Urine output was retrieved hourly for the first 72 h of ICU admission, and the use of RRT was retrieved on days 1 and 90 of the ICU admission. Serum IL-6 levels were measured immediately after the serum levels of creatinine, using the same blood samples originally obtained for the routine clinical measurements. These values were determined using commercially available assay kit (IL-6, Roche Diagnostics K.K., Tokyo, Japan; creatinine, Wako Pure Chemical Industries, Ltd., Osaka, Japan). Serum IL-6 levels from day 1 of the ICU admission were retrieved as the data on admission. Serum creatinine levels were retrieved at baseline, on day 1 of ICU admission, at hospital discharge, and on day 90 after the ICU admission. The baseline creatinine was defined as the lowest documented level within 3 months to 1 week prior to the ICU admission [21]. If this baseline creatinine levels were missing, we calculated the values according to the revised estimated glomerular filtration rate (GFR) from serum creatinine levels adapted for the Japanese population [22], assuming an estimated GFR of 75 mL/min/1.73 m^2^ [5]. For serum creatinine on day 90, data within 7 days of the actual day 90 were considered acceptable. Due to the lack of an established cut off value of IL-6 for evaluating AKI, patients were assigned to three groups according to low, middle, and high admission IL-6 level tertiles.

AKI was diagnosed according to KDIGO criteria. AKI stages on day 1 were determined according to the change in serum creatinine levels from baseline to day 1 (or need for RRT) or urine output within 24 h after the ICU admission [5]. When AKI stages differed between the two criteria, the higher AKI stage was retrieved. In-hospital 90-day persistent AKI was defined as sustention of any kidney injury at 90-day or on hospital discharge in survivors. It was evaluated by the serum creatinine levels at baseline and on day 90 or hospital discharge, or need for RRT according to the KDIGO criteria. Persistent AKI was diagnosed when the creatinine level elevated by 0.3 mg/dL or greater than the baseline, increased 1.5 times or greater than the baseline, or required RRT on day 90 or at the time of hospital discharge. Complete renal recovery was defined as a condition in which the serum creatinine levels on day 90 or hospital discharge decreased to the baseline, and there was no longer a need for RRT [21].

### Statistical analysis

The primary outcome was in-hospital 90-day mortality. The secondary outcome variables were urine output, incidence of persistent anuria for ≥12 h during the first 72 h, and in-hospital 90-day persistent AKI. Pearson’s chi-square test was used to analyze categorical values, while the Kruskal-Wallis test and Dunn’s post hoc test were used to analyze continuous values.

Kaplan-Meier survival curves were plotted for 90-day survival for low, middle, and high IL-6 groups. The log-rank test for trend was used to compare the survival trend until 90-day between the groups. To evaluate the significance of serum levels of IL-6 on ICU admission as an independent risk factor for in-hospital 90-day mortality, incidence of anuria within 72 h, and persistent AKI, Cox regression analysis and multivariate logistic regression analysis including IL-6 (per tertile) and differences in the potential risk factors at baseline among the tertile groups (i.e. age, male sex, pre-existing CKD, APACHE II score, and AKI etiology) were performed [23–25]. To further investigate the association of serum levels of IL-6 and urine output, patients were divided into 10 groups according to the decile, and the cumulative urine outputs for 72 h were compared. Persistent AKI was analyzed in patients who survived for 90 days or survived hospital discharge before 90 days after ICU admission. Since severity scores have been suggested to have a potential association with IL-6 [13, 14, 26], we also performed the multivariate analysis adjusted without APACHE II score.

Data are expressed as median (interquartile range [IQR]) for continuous values and absolute number and percentage for categorical values. Two-tailed *P* values < 0.05 were considered significant. Analyses were performed using JMP Pro 12 (SAS Institute Inc., Cary, NC, USA) or GraphPad Prism 7 statistical software (GraphPad Software, Inc., La Jolla, CA, USA).

## Results

### Baseline characteristics

**Fig. 1:**
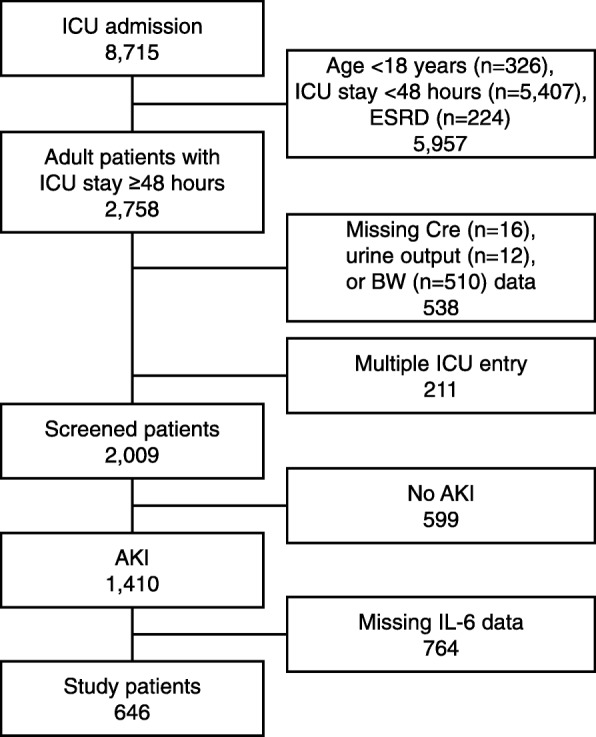
Patient selection process. Out of the 8715 patients who were admitted to the ICU during the study period, 2009 adult patients were screened for eligibility. After the exclusion of non-AKI patients and those without records of ICU admission serum IL-6 levels, 646 AKI patients were enrolled for the analyses. (AKI, acute kidney injury; BW, body weight; Cre, creatinine; ESRD, end-stage renal disease; ICU, intensive care unit; IL, interleukin)

We screened 8715 patients who were admitted to the ICU during the study period; of these, a total of 646 adult patients were eligible for the analyses (Fig. 1). Patients were divided into tertiles based on serum levels of IL-6 on ICU admission (low, 1.5–150.2 pg/mL; middle, 152.0–1168 pg/mL; high, 1189-2,346,310 pg/mL). Sepsis was the most common etiology of AKI (45.5%) followed by cardiovascular disease (19.7%). Patients in the higher IL-6 groups had significantly higher proportions of septic AKI, and lower proportions of cardiovascular disease induced AKI (low vs. middle vs. high; sepsis, *P* < 0.0001; cardiovascular disease, *P* < 0.0001). Patients in the higher IL-6 group had a significantly higher APACHE II score, SOFA score, and AKI stage on day 1 and more frequently required RRT within 24 h after ICU admission (*P* < 0.0001) (Table 1).

**Table 1:** Baseline characteristics and clinical outcomes in patients divided into tertile based on serum levels of interleukin-6 on intensive care unit admission

	Serum levels of interleukin-6			
	Low	Middle	High	*P* value
	(*n* = 215)	(*n* = 216)	(n = 215)	
Characteristic				
Interleukin-6 (range in pg/mL)	1.5–150.2	152.0–1168	1189–2,346,310	
Age, years	65 (52–73)	66 (51–75)	69 (59–76)	0.048
Male sex, n (%)	135 (62.8)	153 (70.8)	137 (63.7)	0.16
Chronic kidney disease, n (%)	43 (20.0)	39 (18.1)	35 (16.3)	0.61
Hypertension, n (%)	92 (42.8)	90 (41.7)	86 (40.0)	0.84
Diabetes mellitus, n (%)	45 (20.9)	48 (22.2)	50 (23.3)	0.84
APACHE II score	26 (20–33)	28 (22–37)	31 (26–39)	< 0.0001
SOFA score	7 (5–10)	9 (6–12)	11 (9–14)	< 0.0001
AKI stage	2 (2–3)	2 (2–3)	3 (3–3)	< 0.0001
RRT within 24 h, n (%)	70 (32.6)	84 (38.9)	159 (74.0)	< 0.0001
Etiology of AKI				
Sepsis	55 (25.6)	89 (41.2)	150 (69.8)	< 0.0001
Cardiovascular disease	56 (26.0)	51 (23.6)	20 (9.3)	< 0.0001
Hypovolemia	17 (7.9)	17 (7.9)	13 (6.0)	0.70
Severe acute pancreatitis	5 (2.3)	21 (9.7)	11 (5.1)	0.0038
Major surgery	10 (4.7)	10 (4.6)	6 (2.8)	0.53
Hepatic failure	13 (6.0)	7 (3.2)	5 (2.3)	0.11
Urinary tract obstruction	5 (2.3)	0 (0.0)	1 (0.5)	0.029
Drug induced	2 (0.9)	0 (0.0)	0 (0.0)	0.13
Other	52 (24.2)	21 (9.7)	9 (4.2)	< 0.0001
Outcome				
Anuria within 72 h, n (%)^α^	30 (16.8)	30 (15.4)	74 (37.6)	< 0.0001
Persistent AKI, n (%)^β^	63 (36.2)	39 (23.8)	34 (23.4)	0.013
In-hospital 90-day mortality, n (%)	41 (19.1)	52 (24.1)	70 (32.6)	0.0050

*APACHE* Acute physiology and chronic health evaluation, *SOFA* Sequential organ failure assessment, *AKI* Acute kidney injury, *RRT* Renal replacement therapy

Data are presented as median and interquartile range for continuous variables. *P* values were calculated using Pearson’s chi-square test or the Kruskal-Wallis test

^α^Analyzed in 571 patients with complete 72-h urine output data. The occurrence of anuria is defined as a cumulative urine output < 50 mL over 12 consecutive hours. ^β^Analyzed in 483 patients who survived for 90 days or survived hospital discharge before 90 days after intensive care unit admission

### Outcome

**Fig. 2:**
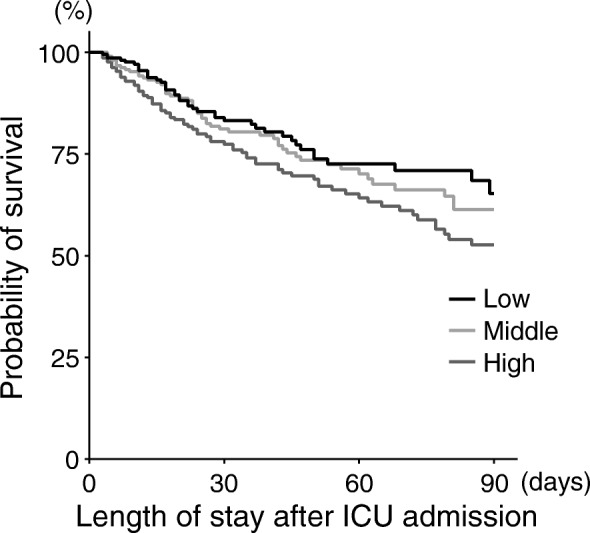
Kaplan-Meier survival curve of each IL-6 group. Higher IL-6 (per tertile) group had lower trend for 90-day survival

In the primary outcome analysis, patients in the high IL-6 group had higher in-hospital 90-day mortality rates (low vs. middle vs. high, *P* = 0.0050) (Table 1) and lower trend for the possibility of survival within 90-day (low vs. middle vs. high, *P* = 0.024) (Fig. 2). In the Cox regression analysis adjusted for APACHE II score, IL-6 had no significant risk for 90-day mortality (Table 2A). But when APACHE II score was excluded from the adjustment, a higher IL-6 level on ICU admission was independently associated with a higher risk of in-hospital 90-day mortality (IL-6 [per tertile], adjusted risk ratio [RR], 1.28; 95% confidence interval [CI], 1.04–1.58, *P* = 0.018) (Table 2B).

**Table 2:** Cox regression analysis of the identification of risk factors of hospital death within 90 days after intensive care unit admission

Variable	Adjusted risk ratio	95% confidence interval	*P* value
A. Analysis adjusted with APACHE II score			
Age	1.00	0.99–1.01	0.96
Male sex	1.43	1.01–2.05	0.042
Interleukin-6 level (per tertile)	1.09	0.88–1.35	0.42
Chronic kidney disease	0.77	0.51–1.12	0.17
APACHE II score	1.06	1.04–1.08	< 0.0001
Etiology			
Cardiovascular disease	reference	–	–
Sepsis	1.16	0.75–1.84	0.51
All other etiologies	1.40	0.89–2.22	0.14
B. Analysis adjusted without APACHE II score			
Age	1.00	0.99–1.01	0.62
Male sex	1.41	1.00–2.02	0.051
Interleukin-6 level (per tertile)	1.28	1.04–1.58	0.018
Chronic kidney disease	0.86	0.57–1.26	0.44
Etiology			
Cardiovascular disease	reference	–	–
Sepsis	0.87	0.57–1.36	0.53
All other etiologies	1.05	0.68–1.66	0.83

*APACHE* Acute physiology and chronic health evaluation

Risk ratio associated with a one-unit change in age, interleukin-6 level (per tertile), and APACHE II score, and associated with positive findings for other variables

**Fig. 3:**
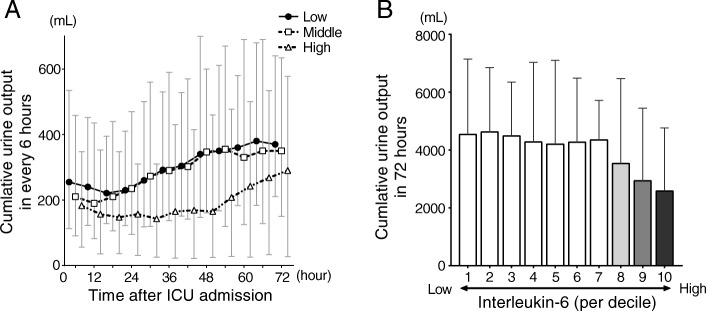
Cumulative urine output within 72 h after ICU admission. **a.** Cumulative urine output of each IL-6 group every 6 h. **b.** Patients were divided into 10 groups according to the decile of IL-6 levels on admission. Higher IL-6 group over the 7th decile had lower urine output. Data are presented as median and interquartile range

In the secondary outcome analysis, patients in the high IL-6 group had lower urine outputs within the first 72 h after ICU admission than the low and middle IL-6 groups (Fig. 3a). The cumulative urine outputs within the first 72 h in the high IL-6 group was significantly lower than the other groups (low vs. middle vs. high, *P* < 0.0001; low vs. middle, *P* = 1.0; low vs. high, *P* < 0.0001; middle vs. high, *P* = 0.0002). When patients were divided into 10 groups according to the decile of serum IL-6 level (1st decile is the lowest serum IL-6 level group and 10th decile is the highest serum IL-6 group), there were no significant differences in the cumulative urine outputs within 72 h after ICU admission among subgroup of 1st to 7th decile groups (*P* = 0.94). Between 8th to 10th decile subgroups, there was a decreasing trend of urine outputs in the higher IL-6 decile groups (*P* = 0.13, minimum IL-6 level of 8th decile group is 1434.8 pg/mL) (Fig. 3b).

Patients in the high IL-6 group had an increased probability of persistent anuria for ≥12 h within the first 72 h of ICU admission (low vs. middle vs. high, *P* < 0.0001) (Table 1). In the multiple logistic regression analysis, a higher IL-6 level (per tertile) was associated with an increased probability of anuria within 72 h of ICU admission with or without adjustments for APACHE II score (IL-6 [per tertile], adjusted with APACHE II score, adjusted OR, 1.58; 95% CI, 1.17–2.13; *P* = 0.0027; adjusted without APACHE II score, adjusted OR, 1.97; 95% CI, 1.49–2.62; *P* < 0.0001) (Table 3A and Table 3B).

**Table 3:** Multivariate logistic regression analysis of the identification of factors predictive of anuria within 72 h after intensive care unit admission

Variable	Adjusted odds ratio	95% confidence interval	*P* value
Age	1.00	0.98–1.01	0.76
A. Analysis adjusted with APACHE II score			
Male sex	0.93	0.59–1.48	0.77
Interleukin-6 level (per tertile)	1.58	1.17–2.13	0.0027
Chronic kidney disease	2.19	1.30–3.68	0.0033
APACHE II score	1.11	1.09–1.14	< 0.0001
Etiology			
Cardiovascular disease	reference	–	–
Sepsis	1.57	0.84–2.94	0.16
All other etiologies	2.06	1.08–3.91	0.028
B. Analysis adjusted without APACHE II score			
Age	1.00	0.99–1.02	0.68
Male sex	0.99	0.64–1.51	0.95
Interleukin-6 level (per tertile)	1.97	1.49–2.62	< 0.0001
Chronic kidney disease	2.31	1.43–3.74	0.0006
Etiology			
Cardiovascular disease	reference	–	–
Sepsis	0.92	0.52–1.64	0.78
All other etiologies	1.16	0.64–2.10	0.62

*APACHE* Acute physiology and chronic health evaluation. Odds ratio associated with a one-unit change in age, interleukin-6 level (per tertile), and APACHE II score, and positive findings for other variables. Data were analyzed in 571 patients with complete 72-h urine output data

Survivors in the high IL-6 group had a lower incidence of persistent AKI on day 90 after ICU admission (low vs. middle vs. high, *P* = 0.013) (Table 1). In the multiple logistic regression analysis with or without adjustment for APACHE II score, higher age and pre-existing CKD were associated with an increased probability of persistent AKI, while a higher IL-6 level was associated with a decreased probability of persistent AKI (IL-6 [per tertile], adjusted with APACHE II score, adjusted OR, 0.67; 95% CI, 0.49–0.91; *P* = 0.0097; adjusted without APACHE II score, adjusted OR, 0.71; 95% CI, 0.53–0.95; *P* = 0.023) (Table 4A and Table 4B).

**Table 4:** Multivariate logistic regression analysis of the identification of factors predictive of persistent acute kidney injury 90 days after intensive care unit admission or hospital discharge in survivors

Variable	Adjusted odds ratio	95% confidence interval	*P* value
A. Analysis adjusted with APACHE II score			
Age	1.02	1.00–1.03	0.010
Male sex	1.01	0.64–1.58	0.98
Interleukin-6 level (per tertile)	0.67	0.49–0.91	0.0097
Chronic kidney disease	6.40	3.78–10.85	< 0.0001
APACHE II score	1.02	1.00–1.05	0.10
Etiology			
Cardiovascular disease	reference	–	–
Sepsis	1.11	0.59–2.10	0.75
All other etiologies	1.22	0.65–2.27	0.53
B. Analysis adjusted without APACHE II score			
Age	1.02	1.01–1.04	0.0058
Male sex	1.00	0.64–1.57	0.99
Interleukin-6 level (per tertile)	0.71	0.53–0.95	0.023
Chronic kidney disease	6.66	3.94–11.26	< 0.0001
Etiology			
Cardiovascular disease	reference	–	–
Sepsis	0.95	0.52–1.76	0.88
All other etiologies	1.06	0.58–1.93	0.84

*APACHE* Acute physiology and chronic health evaluation

Odds ratio associated with a one-unit change in age, interleukin-6 level (per tertile), and APACHE II score, and positive findings for other variables. Data were analyzed in 483 patients who survived 90 days or survived discharge before 90 days after intensive care unit admission

## Discussion

The present study demonstrated that patients with a higher serum level of IL-6 on ICU admission had higher in-hospital 90-day mortality, a lower urine output, and a higher incidence of anuria during the first 72 h of ICU admission. On the contrary, a higher IL-6 level (per tertile) was associated with a lower incidence of 90-day persistent AKI in survivors.

### IL-6 and mortality

Previous reports have shown that the peak serum levels of IL-6 after ICU admission were correlated with organ dysfunction severity when assessed according to the SOFA score organ failure criteria [13]. Similarly, we found that AKI severity was significantly higher in the group with a higher IL-6 level. IL-6 is also known as a predictive biomarker for mortality in critically ill patients [15, 27]. Previous reports of ARF patients showed that the serum levels of IL-6 within 24 h or 48 h from ICU admission were significantly higher in non-survivors than in survivors [18, 19]. We observed a similar relationship, where a higher IL-6 (per tertile) was significantly associated with higher in-hospital 90-day mortality rates.

### IL-6 and urine output

We found that serum levels of IL-6 on ICU admission was associated with lower urine output and the incidence of anuria within the first 72 h of ICU admission. These observations have important implications on the outcomes of patients with AKI as the presence of oliguria or anuria is reportedly associated with increased mortality in ICU patients [3, 28–30]. The association between increased serum IL-6 level and decreased urine output may also guide the decisions to initiate RRT in the early phase of AKI because prolonged oliguria or anuria has been demonstrated as a useful indicator to identify the need for initiating RRT [4]. In fact, a previous report showed that prolonged oliguria was the most common reason for initiating RRT in ICU patients [6].

### Role of IL-6 in renal function recovery

Older age and pre-existing CKD were associated with persistent AKI in the analysis of renal recovery in surviving patients, consistent with the findings of previous reports [31, 32]. In contrast, we found that a higher IL-6 (per tertile) was associated with a higher rate of complete renal recovery in survivors. While age and CKD may be associated with chronic or irreversible background conditions, IL-6 represents acute inflammation in the early phase of critical illness [13, 14, 33], which may be reversible. Previous investigation showed that the survivors of septic AKI patients, who were suggested to have high IL-6 level than non-septic patients [34], had significantly lower creatinine or RRT requirement on discharge than non-septic patients [23]. While our multivariate model found no association of sepsis with renal outcome, high IL-6 levels might be associated with complete renal recovery. In addition, IL-6 is suggested to have kidney-protective role via upregulating anti-oxidative factors and moderating oxidative stress according to several animal studies [35–37]. However, since the long-term renal outcome cannot be evaluated in non-survivors who are likely to be more critical than the survivors, potential effect of IL-6 on renal outcome in critically ill AKI patients is unclear. Further studies are needed to confirm these findings in human subjects.

### Limitations

This study has several limitations. First, this is a single-center retrospective study, and serum IL-6 measurements were made based on the decision of the attending physicians according to the clinical needs. These conditions may introduce selection bias. Second, we could not assess the effect of diuretic agents on urine output; thus, we may have underestimated the AKI diagnosed based on urine output criteria. Third, due to the lack of an established cut off value of IL-6 for predicting mortality in AKI patients, our best option was to conduct the analyzes by categorizing the patients according to the tertiles of the measured values. As a result, the range of the IL-6 values varied among the tertiles. The high IL-6 tertile group presented the widest range of values, reflecting the characteristics of the IL-6 to be overly exaggerated in the patients presenting severe inflammatory response, as observed in previous reports [33, 38]. Fourth, although biomarkers are often used in combination with other parameters to achieve better predictive ability, the focus of the present study was to evaluate the predictive ability of IL-6 as a single biomarker. IL-6 was associated with short- and long-term outcomes in AKI patients, suggesting the potential advantage of using IL-6 as a basis for such combination use.

## Conclusions

Increased serum levels of IL-6 on ICU admission were associated with increased in-hospital 90-day mortality, lower urine output, and higher incidence of anuria within the first 72 h of ICU admission in AKI patients. Serum IL-6 level was also associated with renal recovery in survivors.

## Abbreviations

AKI: Acute kidney injury
ARF: Acute renal failure
ICU: Intensive care unit
IL-6: Interleukin-6
RRT: Renal replacement therapy
